# COVID-19 and medical education: an opportunity to build back better

**DOI:** 10.4314/gmj.v54i4s.18

**Published:** 2020-12

**Authors:** Adwoa Agyei-Nkansah, Patrick Adjei, Kwasi Torpey

**Affiliations:** 1 Department of Medicine and Therapeutics, University of Ghana Medical School, Accra, Ghana; 2 Department of Population, Family and Reproductive Health, University of Ghana School of Public Health, Legon, Ghana

**Keywords:** COVID-19, Medical education, Virtual, Future, Ghana

## Abstract

The coronavirus disease 2019 (COVID-19) outbreak in the Hubei province of China has rapidly transformed into a global pandemic. In response to the first few reported cases of COVID-19, the government of Ghana implemented comprehensive social and public health interventions aimed at containing the disease, albeit its effect on medical education is less clear. Undoubtedly, the COVID-19 has brought changes that may impact the plan of career progression for both students and faculty. Hitherto, medical education had students getting into contact with patients and faculty in a facility setting. Their physical presence in both in-and outpatients' settings has been a tradition of early clinical immersion experiences and the clerkship curriculum. Rotating between departments makes the students potential vectors and victims for COVID-19. COVID-19 has the potential to affect students throughout the educational process. The pandemic has led to a complete paradigm shift in the mode of instruction in a clinical care setting. Inperson training has either been reduced or cancelled in favour of virtual forms of pedagogy. The clinics have also seen a reduction in a variety of surgical and medical cases. This situation may result in potential gaps in their training. Outpatient clinics have transitioned mainly to telemedicine, thus minimizing students' exposure to clinic encounters. Faced with this pandemic, medical educators are finding ways to best ensure rigorous training that will produce competent physicians. This article discusses the status of medical education and the effect of COVID-19 and explores potential future effects in a resource-limited country.

**Funding:**

None declared

## Introduction

The world was awakened to an outbreak of a novel coronavirus (COVID-19) pandemic at the beginning of the year 2020, affecting millions of people. It was found to have started in the Hubei province in China.^1^ The world is interconnected with travel and commerce, which resulted in this epidemic becoming a global pandemic within a few days. This pandemic is primarily transmitted from person to person through respiratory droplets and direct contact. The disease is highly infectious and associated with high mortality, especially among people with underlying health problems. Attention has focused on understanding its pathology and the development of drugs and vaccines to combat the disease. However, is lacking is the assessment of the impact of COVID-19 on medical education in resource-limited settings like Ghana.

By the May 10th, 2020, Ghana had recorded 4,700 confirmed cases of COVID-19. Health experts have predicted that there will be an upsurge in the number of confirmed cases due in part to increased testing leading to the closure of schools, among others.

Resource limited countries like Ghana lack adequate healthcare workforce and this pandemic further exposes the manpower gap prompting the need to prepare future physicians adequately within the context of this pandemic. COVID-19 poses practical and logistic challenges in medical training. Patient safety is key; however, students may become vectors or victims of the disease during training. This article looks at medical education in a limited resource setting in the face of a global pandemic, describing the impact, mitigating factors and effect on future training.

### Medical Education in Ghana

Education plays a pivotal role in the rate of socio-economic, and political development of a country. Effective training is therefore essential in national development. Education helps students progress from one level to another increasing their competency and proficiency as they move towards graduation.^2^ Medical students are to learn all relevant aspects of medicine from preclinical to clinical level to acquire the skill set necessary to function as a doctor. However, the COVID-19 pandemic has disrupted their education and their presence in healthcare facilities may add or pose unnecessary risks for themselves, patients, clinicians and other health personnel as they rotate between departments.

Until recently, medical training was associated with students meeting in physical settings, large class sizes, lecturer-centered activities, tutorials, small group discussions and bedside teachings. Over a decade now, medical educators have been working to convert pedagogy by abolishing or reducing lectures, enhancing training using technology; implementing team-based, and self-directed learning; and promoting professionalism to improve the quality of education.^3^ Sometimes, these changes may undermine key philosophical beliefs on which such innovations are premised, and possibly impact learning outcomes.

The preclinical phase is characterized by didactic lectures, laboratory activities, tutorials, and skills laboratory engagements. Close contact among students and lecturers may occur during this period. The pre-clinical curriculum is relatively easier to migrate online.

The clinical phase is mainly bedside teaching in which students are brought into direct contact with patients. They learn medicine from these exposures under the tutelage of faculty clinicians. Also, during the clinical phase of training, students go on frequent rotations between departments and hospitals. Although this construct leads to scientifically grounded and competent medical practitioners, they are prone to transmitting or getting infected with COVID-19.^4^

A mathematical modelling study by Temime et al, found that R0 for COVID-19 was higher among healthcare staff compared to that of the general population.^5^ This is due to high volume viral inoculation through direct aerosol and droplet exposure compared to viral load of fomiterelated transmission in the public. It is therefore vital to protect the health and safety of this workforce. This informed the idea of students being asked to stay at home to perform some of their academic activities. The daily routine educational activities consist of a mixture of class-based, expert-lecture, tutorials, skill's lab, laboratory work or activity and students' group discussion and independent self-studying. From these activities, expertlecture and tutorials are modifiable to some extent so that both can be done virtually using the internet.

A substantial number of medical students are in the process of preparing for or undertaking assessments that require clinical exposure. Examinations are conducted in the form of essays and multiple-choice questions and bedside clinical skills assessments using the modified objective structure clinical examination (OSCE). In the case of the clinical phase, in addition to the written examinations, clinical skills are assessed using patients. This poses risk to both the student, the examiner, and the patient.

For all educational changes effected during COVID-19, the fundamental consideration is premised on the safety and well-being of the students during their patient-centred learning experience. However, these changes must not interfere with the governance structure of the curriculum to ensure full compliance with accreditation standards.^6^

### Impact of COVID-19

Medical students are eager to complete their course and join the health workforce. The COVID-19 pandemic has however changed the lives of many students, teachers, and parents globally, with many now teaching and learning remotely from home. Although it is still early days, the pandemic may leave long-lasting effects on medical education. According to UNESCO, as of May 6th, 2020, 177 countries had totally shut down their schools affecting over one billion learners globally. Back in the middle of March 2020, the government of Ghana issued guidance to the schools including the medical schools on suspension of in-person training in response to the pandemic. Medical students were required to stay at home. This brought anxiety, fear, and sometimes poor communication and knee jerk responses. Teaching hospitals saw a rapid surge of the disease, shortages of personal protective equipment (PPE), and growing concern for exposures to asymptomatic carriers. The medical students were removed temporarily to protect them from direct patient care.

Many schools have set up courses that are online, many have piloted new experiences like virtual standardized patients, and telemedicine and continue to look for creative ways to impart knowledge and provide clinical service at the same time. Virtual learning has the advantage in its flexibility in time and location.

It has also allowed students to participate in online conferences, which hitherto was not possible due to cost and distance. It also eases the pressure on lecturers in preparing for the lecture including getting to class on time. Additionally, faculty and learners can enjoy discussions and lectures in the comfort of their homes.

Faculty lacking information technology skills must be brought to speed on how to use for example software like Zoom™ and/or Microsoft Teams™. Remote learning requires a different skill set and therefore different training for an educator. Appropriate faculty training and effective coordination is required.

The final year students who were due to complete their rotations and sit for their final examination were hard hit. At the University of Ghana Medical School for instance, their clinic exposure was reduced by three weeks to reduce the risk of contracting the disease given the increasing number of cases in the teaching hospital. This reduced the in person clinical learning opportunities available to them.

In some countries like the UK, the surge of cases led to the suspension of medical and observership students from clinical attachments.^7^ This may impact negatively on their performance and competency. Final year students were also graduated to join the workforce in the fight against COVID-19 through a controlled fast-track programme.^8^ Denmark fast tracked training for their final year students in ventilator therapy and nursing assistance through a digital platform to assist in the management of COVID-19 patients.^9^ The fear in involving relatively inexperienced students is that they might misuse the limited personal protective equipment (PPE)- items thus imposing additional cost burden on the health facilities. In Canada, modifications have been made to the assessment framework by shifting formative and summative assessments; multiple-choice examinations are delivered online and made formative. Clinical assessment for final years were done using online objective structured clinical examination (OSCE) and deferring it for the other students.^9^

History has taught us about medical education during pandemics such as the Severe Acute Respiratory Syndrome (SARS) epidemic in Hong Kong. During the epidemic, medical schools officially cancelled formal ward teaching and delayed their exams. It also provided them the opportunity to integrate IT into teaching methodologies in their medical schools.^10^

Similarly, in Canada, clinical clerkships and electives were suspended for students for almost six weeks. They replaced in-person early clinical exposures and adapted online posting of clinical skills, webinars, and video conferencing.^11^ The challenges of the SARS epidemic led to many resourceful initiatives that revamped medical education. These include online problem-based learning techniques to complete the curricula and are currently being applied in Chinese schools.^10^ Clinical competency also depends on reliable assessment tools that ensure our graduates are prepared to enter residency training with the knowledge and skills to provide safe and effective patient care.

Despite the temptation for innovation, some elements of the curriculum such as skills, work ethics, teamwork and research should not be changed, to preserve quality of education.

These alterations in curriculum should take into cognizance infection prevention, pandemic modelling, and telemedicine, among others. Accrediting agencies will have to join in the adaptation. Accrediting bodies, professional organizations and licensing boards should partner the medical schools during this pandemic response. The changes made will become the new-normal and may be extended to future crisis situations. Again, the pandemic impacts on graduation timeline, and financial liability. The latter though non-academic, must not be overlooked but adequately addressed. While a return to contact patient care and teaching remains highly desirable, a careful look at ways of achieving this aspect of curriculum is vital. Undoubtedly, this undertaking will result in more innovations, flexibility, and experimentation in areas such as problem-based learning, clinical skills education, assessment of students and mentorship. It will be important going forward for medical schools to share their best practices and experiences on medical education during this COVID-19 pandemic.

## Conclusion

Attention in this pandemic has been on care for patients and communities. The need to prepare future physicians has never been urgent as it is now in this global epidemic. The outbreak of severe acute respiratory syndrome coronavirus 2 has disrupted medical education and requires prompt adjustments from medical educators. Notwithstanding the strides made using virtual platforms, challenges remain in equitable access to these services. The development or enhancement of virtual capacity building activities for teachers is paramount. In times of pandemic distress, comfort can always be found as we await creativity for medical education to build back better after the COVID-19 epidemic.
